# Distinct Network Interactions in Particle-Associated and Free-Living Bacterial Communities during a *Microcystis aeruginosa* Bloom in a Plateau Lake

**DOI:** 10.3389/fmicb.2017.01202

**Published:** 2017-06-30

**Authors:** Caiyun Yang, Qi Wang, Paulina N. Simon, Jinyu Liu, Lincong Liu, Xianzhu Dai, Xiaohui Zhang, Jialiang Kuang, Yasuo Igarashi, Xuejun Pan, Feng Luo

**Affiliations:** ^1^Research Center of Bioenergy and Bioremediation, Southwest UniversityChongqing, China; ^2^State Key Laboratory of Biocontrol, Guangdong Key Laboratory of Plant Resources and Conservation of Guangdong Higher Education Institutes, College of Ecology and Evolution, Sun Yat-sen UniversityGuangzhou, China; ^3^Faculty of Environmental Science and Engineering, Kunming University of Science and TechnologyKunming, China

**Keywords:** bloom, *Microcystis aeruginosa*, particle-associated bacteria (PAB), free-living bacteria (FLB), microbial interaction network

## Abstract

Particle-associated bacteria (PAB) and free-living bacteria (FLB) from aquatic environments during phytoplankton blooms differ in their physical distance from algae. Both the interactions within PAB and FLB community fractions and their relationship with the surrounding environmental properties are largely unknown. Here, by using high-throughput sequencing and network-based analyses, we compared the community and network characteristics of PAB and FLB from a plateau lake during a *Microcystis aeruginosa* bloom. Results showed that PAB and FLB differed significantly in diversity, structure and microbial connecting network. PAB communities were characterized by highly similar bacterial community structure in different sites, tighter network connections, important topological roles for the bloom-causing *M. aeruginosa* and Alphaproteobacteria, especially for the potentially nitrogen-fixing (*Pleomorphomonas*) and algicidal bacteria (*Brevundimonas* sp.). FLB communities were sensitive to the detected environmental factors and were characterized by significantly higher bacterial diversity, less connectivity, larger network size and marginal role of *M. aeruginosa*. In both networks, covariation among bacterial taxa was extensive (>88% positive connections), and bacteria potentially affiliated with biogeochemical cycling of nitrogen (i.e., denitrification, nitrogen-fixation and nitrite-oxidization) were important in occupying module hubs, such as *Meganema, Pleomorphomonas*, and *Nitrospira*. These findings highlight the importance of considering microbial network interactions for the understanding of blooms.

## Introduction

Algal blooms could cause severe ecological problems in both marine and fresh water and have both a high frequency and large distribution (Yang C. et al., 2012; Woodhouse et al., 2016). Blooms reduce the water quality (Cai et al., 2013a,b) (e.g., restrict light penetration and increase water viscosity), increase the economic costs of water treatment, change food web dynamics (Turner and Chislock, 2010) and threaten human health (Paerl and Otten, 2013). The relationship between bloom-forming algae and other water microbes has received great attention because they are reported to closely interact; the diversity and community composition of water microbes can be significantly influenced by blooms (Yang C. et al., 2012, 2015; Woodhouse et al., 2016) but can also affect bloom development (Grossart et al., 2005, 2006; Yang C. et al., 2012; Cai et al., 2013a).

Water microbes consist of numerous diverse species (Cai et al., 2013a) and play key roles in biospheric biogeochemical cycling (Woese, 1994). In water ecosystem, bacteria can live either freely or attached to particle (e.g., algae) surfaces (Jasti et al., 2005), with obvious differences in community composition, phylogeny and metabolism (Riemann and Winding, 2001; Grossart et al., 2005; Cao et al., 2015). Free-living bacteria (FLB) are the primary degraders of dissolved organic matter (DOM) in aquatic ecosystems (Karner and Herndl, 1992), and particle-associated bacteria (PAB) are important for the remineralization of both dissolved and particulate organic matter (Tang et al., 2009). PAB are usually associated with colloids and algae (Shao et al., 2014) and are considered algae-associated microbes, particularly during algal blooms (Riemann and Winding, 2001; Cai et al., 2013b), that were shown to have positive or negative effects on algal growth (e.g., algicidal bacteria) in a previous study (Berg et al., 2009). Microbes do not live separately but rather interact with each other to form ecological networks and accomplish ecosystem functions (e.g., material and energy flows, ecosystem stability) (Deng et al., 2015; Shi et al., 2016). Therefore, explaining and predicting the variation in network structure are essential parts of microbial ecology (Zhou et al., 2010) and are useful for guiding practical applications, including biodiversity preservation and ecosystem management. Although the importance of FLB and PAB during algal blooms has been widely addressed (Tuomainen et al., 2006; Cai et al., 2013b; Shao et al., 2014; Cao et al., 2015; Yang C. et al., 2015), the bacterial interactions within the two fractions are still unknown.

Our understanding of microbial interactions in natural environments is limited because most of these microbes are unculturable (Zhou et al., 2010). Culture-independent molecular technology has developed rapidly in recent years and has been acknowledged as a feasible approach to study complex microbial communities. Emerging high-throughput sequencing technologies can provide a comprehensive understanding of the specific microbial species that live and thrive in various environments (Logares et al., 2014), and network-based approaches have been successfully used to evaluate the influence of environmental disturbances on the whole community (Raes and Bork, 2008; Zhou et al., 2010) and analyze microbial interactions in diverse habitats, including marine (Wang Y. et al., 2015), soil (Tu et al., 2015), acid mine drainage (Kuang et al., 2016) and groundwater environments (Deng et al., 2015). However, relevant research on microbial interactions during algal blooms is still limited, especially for the relationship between algae and associated bacteria. Woodhouse et al. (2016) found that microbial communities were closely associated with cyanobacterial composition by using datasets of cyanobacterial cell densities, bacterial community composition and abiotic parameters to demonstrate the interactions of abiotic and biotic factors during cyanobacterial blooms in a shallow ephemeral lake. Nevertheless, the network interaction of PAB or FLB with algae during blooms is still largely unknown.

To fill this gap, in this study, we focused on the following questions during a cyanobacterial bloom in a freshwater lake in the Yunnan-Guizhou plateau: (1) the differences in community composition between PAB and FLB during cyanobacterial blooms; (2) how they respond to environmental fluctuation; and most of all, (3) how the microbes interact in PAB and FLB, particularly considering the role of cyanobacteria in the network interactions.

## Materials and Methods

### Site Description and Sample Collection

**FIGURE 1:**
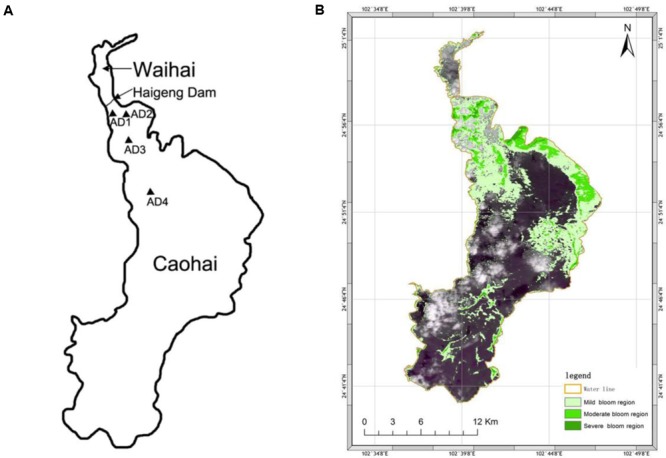
Sampling location **(A)** and bloom area distribution **(B)** of Dianchi Lake during sampling. Sites are marked with triangular icons in Caohai.

The largest plateau lake in China-Dianchi Lake is found in Yunnan Province (Southwest China), with an altitude of 1887 m and a water area of ∼330 km^2^. It is separated into two areas (Caohai and Waihai) by the Haigeng Dam (**Figure 1A**). This lake has become a heavily eutrophied body of water since the 1990s, mainly due to sewage import and phosphorite deposits from the Early Cambrian (Maixing et al., 2003) in this area. Excessive nutrients, strong sunlight and warm temperatures result in the considerable growth of cyanobacteria in this lake. Blooms occur nearly year round and are much more severe in summer and autumn (Li et al., 2003). Specifically, blooms occur regionally in the northwestern side of Waihai with obvious free-floating cyanobacteria and often last more than 8 months (from April to November) annually (Wang et al., 2012).

The dynamics of the bacterial community associated with a cyanobacterial bloom in Dianchi Lake were investigated in this study. Samples were collected in the Waihai of Dianchi Lake at four sites [AD1 (N24.571, E102.394), AD2 (N24.571, E102.385), AD3 (N 24.555, E 102.394), and AD4 (N 24.526, E 102.405)] during a cyanobacterial bloom on October 16th, 2015 (**Figure 1A**). AD1 and AD2 were taken from an area where blooms occur frequently, while AD3 and AD4 were close to and away from this bloom area, respectively. Water samples (2 L for each sample) were collected in three replicates from each site within 20 m^2^ (depth of 0.5 m) using autoclaved polypropylene sampling vials. Immediately after collection, samples for phytoplankton species identification and counts were preserved using 1.5% Lugol’s iodine solution (Lack, 1971), and a separate sample was fixed with PBS-buffered formaldehyde (2% final concentration) for bacteria enumeration. The 5-μm filters combined with a filtration volume strategy were used to separate the PAB and FLB. Specifically, water was filtered through 5-μm filters (Millipore, United States) to collect PAB, and the water volume (60–85 ml) was chosen to avoid retention of FLB (the filtration process should be smooth and rapid). Although the single bloom-causing algae (*M aeruginosa*, cell size ∼1–2 μm) could pass through, they usually aggregate together with other cells in natural water and are retained on the 5-μm filter. Thus, the PAB community contained both algae and bacteria that were attached to algae. The FLB and small amounts of single *M*. *aeruginosa* in the filtrate were collected with 0.22-μm filters (Millipore, United States). In total, 24 samples (12 for PAB, 12 for FLB) were collected, and all filters were stored at -70°C until nucleic acid extractions.

### Water Characterization

The pH was measured using a handheld pH meter (Model IQ150, IQ Scientific Instruments, United States). Dissolved oxygen (DO) and temperature were measured using a handheld DO meter (AM-39-Sensotechnik Meinsberg, Germany). Turbidity and suspended particles were measured using a MultiDirect spectrophotometer (Lovibond, Germany).

Bacteria enumeration was performed using epifluorescence microscopy using DAPI stain. Samples fixed with PBS-buffered formaldehyde were stained with 4′,6-diamidino-2-phenylindole fluorochrome (DAPI; final concentration of 5 μg ml^-1^) (Porter and Feig, 1980); bacterial cells were counted using epifluorescence microscopy and mean bacterial volumes were determined using measurements of 30–100 cells using photographic slides; 15 random visual fields were analyzed for each sample. Phytoplankton cells preserved with Lugol’s iodine solution were counted under an inverted microscope with a hemocytometer (Lund et al., 1958).

The concentrations of NH_4_-N, NO_2_-N, NO_3_-N, total N and soluble phosphate were measured using Nessler’s reagent spectrophotometry (Chen and Hu, 2011), spectrophotometry, phenol disulfonic acid spectrophotometry, alkaline potassium persulfate digestion ultraviolet spectrophotometry and ammonium molybdate spectrophotometry using a UV-2600 UV-VIS spectrophotometer (State Environmental Protection Administration of China, 1989). Chemical oxygen demand (COD) was measured using a Lovibond RD 125 COD Reactor (Germany) and an ET99732 multi-parameter water quality analyzer (Lovibond, Germany) with an ET99955 kit (determination range: 0–150 mg/L O_2_) according to the operation manual. The eutrophication index (EI) was calculated as EI = DIN × DIP × COD × 10^6^/4500 (Yang C. et al., 2015), where DIN is the dissolved inorganic nitrogen (sum of NO_3_-N, NO_2_-N, and NH_4_-N) content in mg/L; DIP is the dissolved inorganic phosphorus content (soluble phosphorus PO_4_-P) in mg/L; and COD is the chemical oxygen demand in mg/L.

### Microbial Community Analysis

#### DNA Extraction and PCR Amplification

The microbial cells were scraped from the collected filters using sodium phosphate buffer. Total DNA was extracted using a FastDNA Spin Kit for Soil (MP Biomedicals, United States) and detected using 1% agarose gel electrophoresis. The V3–V4 region of the microbial 16S rRNA gene was amplified with primers 338F (5′-barcode-ACT CCT ACG GGA GGC AGC AG-3′) and 806R (5′-GGA CTA CHV GGG TWT CTA AT-3′). Amplicons were purified using an AxyPrep^TM^ DNA Gel Extraction Kit (AXYGEN, United States) and quantified using a QuantiFluor-ST Fluorometer (Promega, United States), where the barcode is an eight-base sequence unique to each sample. PCR reactions were performed in triplicate in a 20-μL mixture containing 4 μL of 5 × FastPfu Buffer, 2 μL of 2.5 mM dNTPs, 0.8 μL of each primer (5 μM), 0.4 μL of FastPfu Polymerase, and 10 ng of template DNA.

#### Illumina MiSeq Sequencing

Equimolar amounts of purified amplicons were pooled and paired-end sequences (2 × 250bp) were retrieved from an Illumina MiSeq platform at Majorbio Bio-Pharm Technology Co., Ltd. (Shanghai, China) using a TruSeq^TM^ DNA Sample Prep Kit (Illumina, United States). Raw reads of this study have been deposited into the NCBI Sequence Read Archive using accession No. PRJNA341818.

#### Processing of High-Throughput Sequencing Data

Fastq files of raw reads were demultiplexed and quality-filtered using QIIME (version 1.9.1) using the following criteria: (i) the 300 bp reads were truncated at any site with an average quality score < 20 over a 50-bp sliding window, discarding the truncated reads that were shorter than 50 bp; (ii) exact barcode matching, 2-nucleotide mismatch in primer matching and reads containing ambiguous characters were removed; (iii) only sequences with overlap longer than 10 bp were assembled according to their overlap sequence. Reads that could not be assembled were discarded.

After deleting unqualified sequences, the identification of operational taxonomic units (OTUs) at a 97% similarity level were performed using UPARSE (version 7.1)^1^; chimeric sequences were identified and removed using UCHIME. Taxonomic assignment was performed using RDP Classifier^2^ against the SILVA (SSU123)16S rRNA database using a confidence threshold of 70% (Amato et al., 2013). The alpha-diversities, including Shannon index (Hill, 1973), Chao richness estimator (Chao, 1984) and Good’s coverage (Esty, 1986) were calculated using Mothur (version: 1.30.1) (Schloss et al., 2011). The number of reads ranged from 31,736 to 99,733 in our samples, and 31,736 sequences were randomly resampled for subsequent statistical analyses. Additionally, some analyses were conducted based on the 4512 resampled sequences that were not affiliated with cyanophyta.

#### Network Construction and Analysis

The network analyses were performed using the online Molecular Ecological Network Approach Pipeline^3^, which implements a random matrix theory (RMT) for threshold identification (Deng et al., 2012). The RMT-based approach is a widely used and robust tool for network construction and has been successfully used to construct various networks, including gene regulatory networks (Zhou et al., 2010; Lin et al., 2011, 2013), functional molecular ecological networks (Zhou et al., 2010) and phylogenetic molecular ecological networks (Zhou et al., 2011; Tu et al., 2016). To construct reliable co-occurrence ecological networks, the RMT-based network method was used (Deng et al., 2015), and only OTUs observed in at least 8 of the 12 samples were used for subsequent network analyses. Specifically, the validated pairwise correlations between OTUs were determined based on a Pearson correlation coefficient cutoff of 0.9 and 0.89 that was automatically identified based on RMT for PAB and FLB, respectively, and then was used for network construction. Modules were detected by fast greedy modularity optimization (Clauset et al., 2010), and the reconstructed ecological networks were then visualized using Cytoscape (Shannon et al., 2003). The node connectivity was determined based on both within-module connectivity (Zi) and among-module connectivity (Pi) (Guimerà and Amaral, 2005), which were then used to classify nodes into four topological roles: module hubs (highly connected nodes within modules with Zi > 2.5), network hubs (highly connected nodes within the entire network with Zi > 2.5 and Pi > 0.62), connectors (nodes that connect modules with Pi > 0.62), and peripherals (nodes connected in modules with few outside connections with Zi < 2.5 and Pi < 0.62) (Zhou et al., 2010; Deng et al., 2012).

### Statistical Analyses

Most statistical analyses were performed using the vegan (Oksanen et al., 2013) and agricolae packages (Mendiburu, 2014) in R (R Core Team, 2015). Dissimilarity tests were conducted based on the Bray-Curtis dissimilarity index and two non-parametric tests [i.e., permutational multivariate analysis of variance (Adonis) and multiple-response permutation procedure (MRPP)]. Principal component analysis (PCA) was used to display and compare the patterns of microbial communities among different samples, and canonical correspondence analysis (CCA) was used to link variations in microbial communities to environmental properties. Variation partitioning analysis (VPA) was conducted to examine the contribution of environmental factors in influencing microbial communities according to the CCA analysis. All significant differences were defined as a *p*-value of < 0.05.

## Results

### Water Chemical, Physical, and Biotic Characteristics

A large bloom dominated the northern portion of Dianchi Lake during the sampling period (**Figure 1**). It was a *Microcystis* sp. bloom, according to microscopic examination (*Microcystis* cells represented 98.27–100% of the total algae, Supplementary Table S1). **Table 1** shows the measured environmental properties of all samples. The water pH was much higher than 7 and even reached 10.19 because of the algal bloom (Yang C. et al., 2015). The lake exhibited eutrophication (EI > 1), which was much more severe in AD1 and AD2 (**Table 1**). The soluble phosphorus (PO_4_-P) concentrations were very low; most were below the detectable limit (0.01 mg/L) of our method. Furthermore, the bacterial density was much higher in AD1 and AD2 and was positively correlated at all sites with NO_3_-N (spearman correlation, *p* < 0.01), NO_2_-N (*p* = 0.03), EI (*p* = 0.04) and pH (*p* = 0.035). Additionally, algal density was positively correlated with NO_2_-N (*p* = 0.02), NO_3_-N (*p* = 0.04), COD (*p* < 0.01), and EI (*p* < 0.01) at all sites.

**Table 1:** Environmental and biological properties of samples.

Sample	T	DO	pH	Tur	SP	NO_3_-N	NO_2_-N	NH_4_-N	TN	PO_4_-P	COD	EI	Bac	Algae
AD1-1	18.6	11	9.68	29	36	0.43	0.059	0.231	2.27	<0.01	34.7	55.44	202.95	73.72
AD1-2	18.7	10.9	9.6	28	39	0.46	0.06	0.241	2.85	<0.01	29.30	49.55	241.31	62.34
AD1-3	18.9	10.9	9.71	33	39	0.42	0.058	0.207	3.16	<0.01	26.6	40.42	325.40	56.49
AD2-1	19.1	11.1	10.03	62	69	0.11	0.01	0.393	4.22	<0.01	27.0	30.75	281.66	57.39
AD2-2	19.1	10.7	10.14	53	65	0.13	0.008	0.351	3.46	<0.01	17.0	18.47	192.57	36.17
AD2-3	18.9	10.9	10.17	62	72	0.12	0.009	0.394	4.23	<0.01	21.7	25.22	188.57	46.17
AD3-1	18.1	11.3	9.84	44	51	0.1	0.029	0.27	2.78	<0.01	4.4	3.90	75.75	9.36
AD3-2	18.3	11.1	9.92	45	49	0.1	0.029	0.202	1.99	0.014	16.7	17.20	22.49	35.53
AD3-3	18.5	11.1	9.92	52	53	0.11	0.028	0.299	3.86	<0.01	50.3	48.82	17.78	106.9
AD4-1	19.9	9	10.1	30	34	0.05	<0.003	0.134	1.36	<0.01	4.3	1.77	27.29	9.20
AD4-2	19.1	9.4	10.19	39	39	0.04	<0.003	0.226	2.03	0.011	17.3	11.22	6.79	36.70
AD4-3	18.9	9.5	10.19	40	40	0.06	<0.003	0.247	2.83	<0.01	3.7	2.49	11.25	7.77

*T, temperature, °C; DO, dissolved oxygen, mg/L; Tur, turbidity; SP, suspended particle, mg/L; NO_*3*_-N, nitrate nitrogen, mg/L; NO_*2*_-N, nitrite nitrogen, mg/L, < 0.003 is below detectable limit; NH_*4*_-N, ammonia nitrogen, mg/L; TN, total nitrogen, mg/L; PO_*4*_-P, soluble phosphorus, mg/L, < 0.01 is below detectable limit; COD, chemical oxygen demand, mg/L; EI, eutrophication index; Bac, bacterial density, × 10^*8*^ cells/L; Algae, algal density, × 10^*7*^ cells/L.*

### Microbial Diversity and Community Composition

In this study, a total of 1,638,726 high-quality sequences remained after processing and singleton removal (total 118,108 sequences), and 1633 OTUs (PAB: 1224 OTUs and FLB: 1574 OTUs) were identified at a 97% similarity level. The sequencing depth was sufficient to represent entire whole microbial community because the sequence Good’s coverage was higher than 99.5%. These OTUs belonged to 37 phyla, 54 classes, 104 orders, 182 families and 275 genera (**Supplementary Figure S1a**). Most taxa were shared between the microbial communities of PAB and FLB, and more unique taxa were found in the community of FLB at all five taxonomic levels (**Supplementary Figure S1b**).

A randomly selected subset of 31,736 sequences per sample was obtained based on total qualified sequences (without singletons) using the subsample function of Mothur (version v.1.30.1) (Schloss et al., 2011). The predominant phyla (**Supplementary Figure S2** and Table S3) of the PAB communities were affiliated with Cyanobacteria (73.4 ± 12.2%), Proteobacteria (17.7 ± 8.8%) and Bacteroidetes (5.3 ± 2.6 %), whereas Actinobacteria (36.1 ± 5.9%), Proteobacteria (26 ± 3.9%), and Bacteroidetes (21.3 ± 6.4%) were the most abundant phyla in the FLB communities. *M. aeruginosa* was the dominant species in PAB (Supplementary Table S3) and accounted for 92.97–99.04% of Cyanobacteria, indicating that *M. aeruginosa* was the bloom-causing species.

**FIGURE 2:**
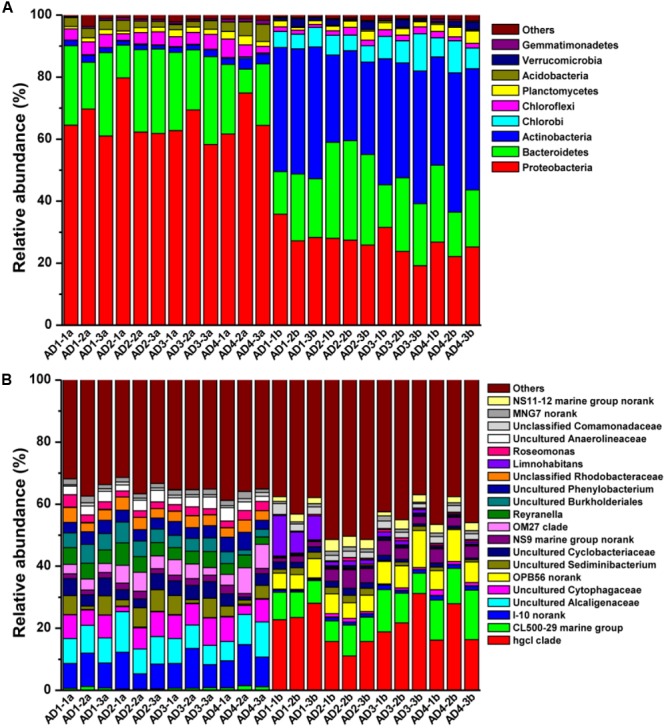
Distribution of bacterial taxa at phylum **(A)** and genus **(B)** levels based on sequence data without cyanobacteria (Supplementary Table S2-resample 2). Sample names with a and b represent PAB and FLB, respectively.

A comparison of PAB and FLB composition based on algae-free data (Supplementary Table S2-Resampling 2) was performed to investigate the difference in bacterial community without cyanobacteria. As shown in **Figure 2A**, the predominant PAB phyla were affiliated with Proteobacteria (65.88 ± 6.3%) and Bacteroidetes (21.24 ± 6.9%), whereas Proteobacteria (26.77 ± 4.3%), Actinobacteria (37.43 ± 5.7%) and Bacteroidetes (21.8 ± 6.5%) were the most abundant FLB phyla. The relative abundance of 14 phyla (43.75%) significantly differed in the two bacterial communities, as shown in Supplementary Table S4. Down to the genus level, three uncultured genera from the phylogenetic cluster I-10 (9.28 ± 2.4%, belongs to Rhodospirillales), Alcaligenaceae (8.68 ± 2.0%) and Cytophagaceae (6.47 ± 2.2%) were the most dominant genera in the PAB community, whereas *hgcI clade, CL500-29 marine group* and uncultured OPB56 from Chlorobiales dominated the FLB community (**Figure 2B**). The physiological characteristics were unclear for these most dominant taxa in PAB and FLB; e.g., the HgcI cluster is only known to be a widespread and typical bacteria group in freshwater (Frank et al., 2000).

Compared to the FLB community, 76 (19.54%) and 103 (26.48%) taxa at the genus level had a significantly higher and lower (*p* < 0.05) relative abundance in PAB, respectively. Most of these taxa could not be assigned to known genera, other than the 86 genera shown in Supplementary Table S5. Additionally, the Shannon diversities of FLB communities were significantly higher (*T*-test, *p* < 0.01) than those of PAB, according to the datasets with or without Cyanobacteria (FLB: 4.87 ± 0.24/4.72 ± 0.23; PAB: 1.85 ± 0.62/4.25 ± 0.16; Shannon diversity with / without *Cyanobacteria*).

### Differences in Whole Community Structure between PAB and FLB

**FIGURE 3:**
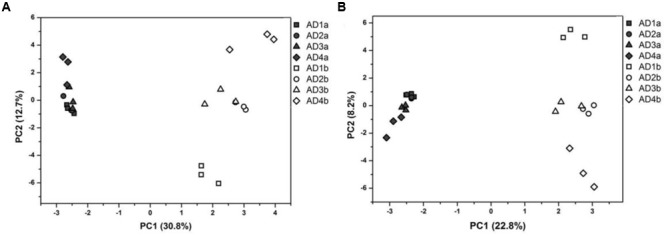
Principal component analyses of total bacteria **(A)** and bacteria without cyanobacteria **(B)**. Filled symbols represent PAB, and hollow symbols represent FLB, respectively.

Dissimilarity tests based on both MRPP (δ = 0.23, *P* < 0.01) and Adonis (*R*^2^ = 0.869, *P* < 0.01) showed significant differences between the PAB and FLB communities. The PCA plots suggested that the whole community structure of PAB was markedly different from that of FLB (**Figure 3A**), and this pattern was consistent even when the cyanobacterial sequences, which were much more abundant in PAB communities, were removed (**Figure 3B**).

### Relationship between Bacterial Communities and Environmental Properties

**FIGURE 4:**
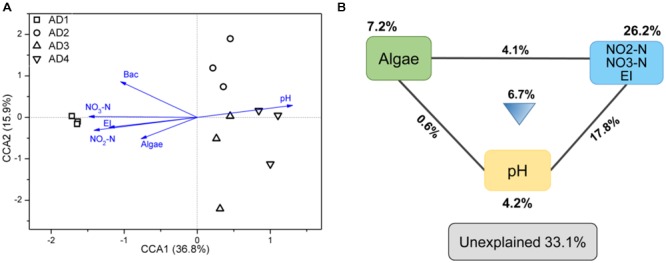
Canonical correspondence analysis (CCA) of FLB with selected environmental variables **(A)** and partial CCA-based variation partitioning analysis (VPA) **(B)**. Selected environmental variables include pH, nitrate nitrogen (NO_3_-N), nitrite nitrogen (NO_2_-N), bacterial density (Bac), algae, eutrophication index (EI). Each diagram represents biological variation partitioned into the relative effects of each factor or a combination of factors.

In this study, 13 environmental variables were used to test their correlation with the two communities (PAB and FLB) (**Table 2**). The results showed that no detected variable had significant (*P* < 0.05) effects on the microbial PAB communities, whereas six environmental variables, including pH, NO_3_-N, NO_2_-N, EI, bacterial density and algae density, significantly correlated with the FLB communities (*P*-values were less than 0.05 based on both Bray-Curtis and Euclidean distances). Because FLB communities were greatly correlated with these environmental properties, CCA was further performed to link the variation of FLB communities to these seven selected variables. The CCA ordination plot revealed that the first two canonical axes explained 36.9 and 17.5% of the constrained variations, respectively (**Figure 4A**). Samples in AD1 were characterized by higher algal density, NO_3_-N, NO_2_-N, EI and bacterial density, and these variables showed positive correlations with each other. AD3 and AD4 were characterized by higher pH. Additionally, samples in AD2 had weak relevance with these environmental properties but had higher Tur, SP, NH_4_-N, and TN that were not counted in CCA. Because of the importance of nutrient properties (i.e., NO_3_-N, NO_2_-N, EI), algae and pH in this CCA result, the VPA based on partial CCA was subsequently conducted using these selected variables. VPA revealed that nutrient properties, algae and pH and their interactions explained 26.2, 7.2, 4.2, and 29.2% of the variation in the FLB communities, respectively, leaving 33.1% unexplained (**Figure 4B**).

**Table 2:** Correlation between factors and bacterial communities based on Adonis (permutational multivariate analysis of variance using distance matrices).

Factors	PAB		FLB	
	Bray-Curtis	Euclidean	Bray-Curtis	Euclidean
	*F*	*F*	*F*	*F*
Temperature (°C)	1.7	2.1	0.8	0.6
Dissolved oxygen (mg/L)	3.2	3.6	1.9	1.5
pH	1.4	1.3	4.4ˆ**	3.7ˆ**
Turbidity	0.3	0	2.1	1.7
Suspended particle (mg/L)	0.5	0.2	2	1.7
NO_3_-N (mg/L)	1.6	1.6	5.6ˆ**	5^∗∗^
NO_2_-N (mg/L)	2.2	2.2	5.3ˆ**	4.6ˆ**
NH_4_-N (mg/L)	0.1	0.1	1.4	1.1
Total nitrogen (mg/L)	0.4	0.1	1.1	0.8
EI	1	0.77	4.5ˆ**	4.6ˆ**
Bacterial density (× 10^8^ cells/L)	0.9	0.8	3.3ˆ**	3.3ˆ*
Algae(× 10^7^ cells/L)	0.1	0.1	3.1ˆ**	4^∗∗^

*^∗^0.01 ≤*p* < 0.05; ^∗∗^*p* < 0.01.*

### Distinct Characteristics and of PAB and FLB Networks

**FIGURE 5:**
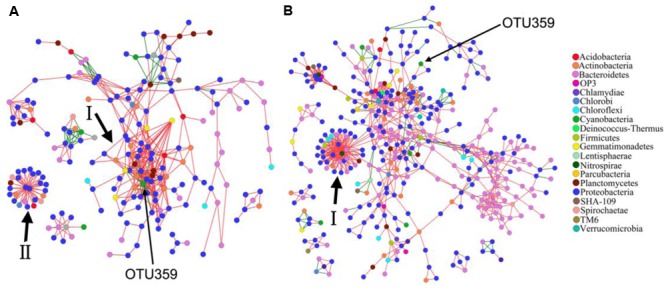
Network interaction of OTUs in PAB **(A)** and FLB **(B)**. Node of bloom-causing *M. aeruginosa* OTU 359 is marked with long arrows, and major modules are marked with short arrows and Roman numerals. Red lines indicate positive interactions, and green lines indicate negative interactions.

In this study, a total of 913 and 1201 OTUs remained for PAB and FLB network construction, respectively (**Figure 5** and **Table 3**), after removing OTUs that did not contain sequence reads in either the PAB or the FLB communities. The sizes (i.e., number of node) of these two networks differed (PAB: 222 and FLB: 480), with only shared 130 nodes (22.7%). Overall, nodes tended to be positively correlated (red lines) rather than negatively correlated (green lines), and positive correlations accounted for 88.2 and 88.8% in the PAB and FLB networks, respectively. The overall topologies (**Table 3**) revealed a more complex PAB network, with a higher average connectivity (avgK) and significantly tighter connections between nodes with a smaller average path length (GD, *p* < 0.01). Similar hierarchical properties in PAB and FLB networks were observed, with a close average clustering coefficients (avgCC).

**Table 3:** Major topological properties of empirical and random MENs of PAB and FLB.

	Network properties	Community	
		PAB	FLB
Empirical networks	No. of original OTU	913	1201
	No. of node	222	480
	No. of link	536	886
	Modularity (no. of modules)^∗∗^	0.675 (21)	0.806 (54)
	Avg connectivity (avgK)^a^	4.829	3.692
	Avg geodesic distance (GD)^b,∗∗^	5.257	7.171
	Avg clustering coefficient (avgCC)^c^	0.2	0.206
Random networks	Avg Modularity (M)	0.411 ± 0.008	0.537 ± 0.005
	Avg geodesic distance (GD)	3.374 ± 0.050	4.311 ± 0.047
	Avg clustering coefficient (avgCC)	0.067 ± 0.012	0.018 ± 0.004

*^a^avgK, avg connectivity, higher avgK means a more complex network. ^b^GD, geodesic distance; smaller GD means that all of the nodes in the network are closer. ^c^avgCC, avg clustering coefficient, higher avgCC (e.g., 1) means a node is fully connected with its neighbors; a value close to 0 means hardly any connections with neighbors. ^∗∗^Significant difference (*p* < 0.01) between PAB and FLB.*

**FIGURE 6:**
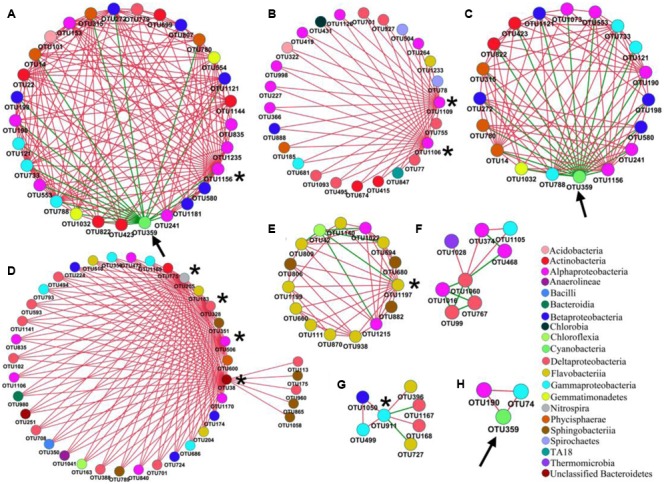
Network interactions between nodes of interest and their nearest neighbors in PAB **(A–C)** and FLB **(D–H)**. Interactions of **(A,B,D–G)** are centered with module hubs (marked with asterisk). Interactions of **(C,H)** are centered with *M. aeruginosa* OTU 359 (marked with arrow).

For the PAB network (**Figure 5A**), a total of 21 modules were generated, and the nodes were affiliated with 12 phyla, mainly from Proteobacteria (60.4%), Bacteroidetes (14.9%) and Actinobacteria (7.7%). The 222 nodes were connected by 536 links (i.e., 473 positive and 63 negative interactions). The Alphaproteobacteria was the largest group (21.6%) in the PAB network, and three module hubs (OTUs 1106- *Meganema*, 1109- *Pleomorphomonas* and 1156- *Brevundimonas* sp., **Figures 6A,B**) of the PAB network were all affiliated with Alphaproteobacteria (**Table 4**), with a Zi value larger than 2.5. Node OTU1156 had the highest connectivity in the PAB network, with 28 positively correlated nodes and 1 negatively correlated node (**Figure 6A**). There was no connector in the PAB network. Compared to the PAB network, the FLB network was much larger, with 54 modules (**Figure 5B**) and 480 nodes connected by 886 links (i.e., 787 positive and 99 negative interactions). The nodes were from 21 phyla and were dominated by *Proteobacteria* (46%), *Bacteroidetes* (30%) and *Actinobacteria* (6.9%). There were three connectors (Pi > 0.62) and seven module hubs (Zi > 2.5), as shown in **Table 4**. Module hub OTU38 (Bacteroidetes) had highest connectivity in the FLB network, with 36 positively correlated nodes (**Figures 5B-I, 6D**). For these module hubs and connectors, only five genera were identified (**Table 4**): *Nitrospira, Fluviicola, CL500*-29 *marine group, Legionella, Crocinitomix*.

**Table 4:** Classification and relative abundance of module hub and connector nodes in PAB and FLB networks.

Classification of nod.	Group	OTU	Class	Taxonomic identification^a^	Ave. Rela. Abun. (%)
Module hub	PAB	OTU1106	Alphaproteobacteria	*Meganema*	0.007
		OTU1109	Alphaproteobacteria	*Pleomorphomonas*	0.006
		OTU1156	Alphaproteobacteria	*Brevundimonas* sp.	0.063
	FLB	OTU38	Unclassified Bacteroidetes	Bacteroidetes	0.007
		OTU506	Alphaproteobacteria	Alphaproteobacteria	0.007
		OTU328	Deltaproteobacteria	Alphaproteobacteria	0.007
		OTU205	Nitrospira	*Nitrospira*	0.006
		OTU1197	Flavobacteriia	*Fluviicola*	0.122
		OTU911	Gammaproteobacteria	Gammaproteobacteria	0.006
		OTU1060	Deltaproteobacteria	Desulfuromonadales	0.006
Connector	FLB	OTU312	Actinobacteria	*CL500-29 marine group*	0.014
		OTU742	Gammaproteobacteria	*Legionella*	0.026
		OTU1044	Flavobacteriia	*Crocinitomix*	0.012

*^a^The classification of OTUs to the least known taxonomic hierarchy.*

### Network-Based Associations between Bacterial Species and *M. aeruginosa*

The network topology was used to explore the correlations among bloom-causing algae and algae-associated microorganisms. In our dataset, OTU359 was the bloom-causing *M. aeruginosa* species, with a relative abundance ranging from 48.9 to 86.1% in PAB (Supplementary Table S3) and a distinct, characteristic connection in the 2 networks, as shown in **Figure 5** (marked with long arrows) and **Figures 6E,H** (marked with arrows). In the PAB network, OTU359 had only negative connections and among its 18 neighbor nodes (**Table 5**), only 8 genera were identified: *SM1A02* (Phycisphaerae), *Brevundimonas, Silanimonas, CL500-29 marine group* (Acidimicrobiales), *Gemmatimonas, Phenylobacterium, Reyranella*, and *Tepidicella.* In contrast, the OTU 359 connected with only 2 nodes (OTU190 and OTU74) with positive links (**Figure 6** and **Table 5**) in FLB network, and one was identified as a genus of *Rheinheimera*.

**Table 5:** Classification and relative abundance of nodes connected to OTU359 (bloom-causing *M. aeruginosa*).

Group	OTU	Class	Taxonomic identification	Ave. Rela. Abun. (%)
PAB	OTU423	Actinobacteria	*CL500-29 marine group*	0.071
	OTU822	Actinobacteria	*Acidimicrobiales*	0.047
	OTU1032	Gemmatimonadetes	*Gemmatimonas*	0.079
	OTU14	Phycisphaerae	*SM1A02*	0.022
	OTU315	Phycisphaerae	*SM1A02*	0.021
	OTU780	Phycisphaerae	*SM1A02*	0.049
	OTU1156	Alphaproteobacteria	*Brevundimonas* sp.	0.063
	OTU553	Alphaproteobacteria	*Phenylobacterium*	1.044
	OTU190	Alphaproteobacteria	*Rhodobacteraceae*	0.920
	OTU241	Alphaproteobacteria	*I-10*	0.208
	OTU1073	Alphaproteobacteria	*Reyranella*	1.162
	OTU198	Betaproteobacteria	*Alcaligenaceae*	2.335
	OTU1121	Betaproteobacteria	*Comamonadaceae*	0.062
	OTU580	Betaproteobacteria	*Tepidicella*	0.110
	OTU272	Betaproteobacteria	*Nitrosomonadaceae*	0.231
	OTU121	Gammaproteobacteria	*Xanthomonadaceae*	0.387
	OTU733	Gammaproteobacteria	*Silanimonas*	0.293
	OTU788	Gammaproteobacteria	*Silanimonas*	0.279
FLB	OTU74	Gammaproteobacteria	*Rheinheimera*	0.032
	OTU190	Alphaproteobacteria	*Rhodobacteraceae*	0.083

## Discussion

In this study, we found significant differences between PAB and FLB communities in terms of diversity, taxonomic composition and microbial network interactions. The greater difference observed between fractions (i.e., PAB vs. FLB) rather than sites was consistent with previous bacterial community studies during a diatom bloom (Riemann and Winding, 2001; Rieck et al., 2015). Although PAB community structure was quite conserved across the environmental changes in surrounding water, the FLB community was significantly correlated with the measured environmental variables (**Table 2**).

Cyanobacteria release considerable amounts of organic matters to surroundings and these organic substrates can attract a lot of heterotrophic bacteria into the phycosphere (Cai et al., 2013a). Because the compositions of these exudates from different algal species are distinct (Sapp et al., 2007; Paver et al., 2013), the divergent availability and physicochemical characteristics of these substrates result in different bacterial communities in the specific algal phycosphere (Louati et al., 2015). Only bacteria from the aquatic system adapting to this microenvironment live and persist in the phycosphere. When *M. aeruginosa* dominated the phytoplankton of Dianchi Lake, phycosphere selection theory could well explain the stability and lower diversity of PAB in this conserved phycosphere (Louati et al., 2015). The regional water environment fluctuated in different sites, including organic and inorganic matter (**Table 1**). This could lead to significant differences in FLB communities from the four sites.

For those genera whose relative abundance significantly changed between PAB and FLB communities, their most likely ecological physiological functions were investigated based on taxonomy according to the literature. The relative abundance of bacteria affiliating with nitrogen-fixing taxa *Candidatus Competibacter* (Mcilroy et al., 2014), *Pleomorphomonas* (Xie and Yokota, 2005; Im et al., 2006) and *Bradyrhizobium* (Kaneko et al., 2002) significantly increased in the PAB community, while the abundance of a potential nitrogen fixer affiliating with *Paenibacillus* (von der Weid et al., 2002) significantly increased in FLB community. Inorganic N is essential and beneficial for cyanobacterial growth; also algal density was found significantly positive correlations with the concentrations of NO_2_-N and NO_3_-N in this study. In our previous study, the *nifH* gene (nitrogen fixation) intensity was also found increased when algal bloom of *Akashiwo sanguinea* occurred (Yang et al., 2016). Nitrogen-fixing microorganisms and algae can form symbiotic relationships, e.g., azotobacteria provided cyanobacteria with inorganic N in exchange for organic exudates from algae (Lili et al., 2014) and Xu et al. (2016) found the cell density of *Bradyrhizobium japonicum* significantly increased when co-cultured with transgenic *Chlamydomonas reinhardtii* hemHc-lbac. This mutually beneficial relationship may have resulted in a significantly higher relative abundance of nitrogen-fixing bacteria in the PAB community than in FLB.

For the bacteria known for methane-utilizing, methanotrophy genera, the relative abundance of bacteria affiliating with methanotrophy genera *Methylomonas* (Koh et al., 1993), *LD28 freshwater group* (Salcher et al., 2015) and *Candidatus Methylacidiphilum* (Khadem et al., 2012), and methanotrophic genus *Methylocaldum* (Murrell, 2010) significantly increased in the FLB community, while the abundance of one filamentous methane oxidizer affiliating with *Crenothrix* (Stoecker et al., 2006) significantly increased in the PAB community. The relative abundance of some potentially algicidal bacteria of *M. aeruginosa* also significantly changed, e.g., *Aquimonas* (Cui et al., 2012) was more abundant in PAB community, and *Pedobacter* (Yang et al., 2012) and *Aeromonas* (Liu et al., 2012) were more so in FLB community. For potentially organic matter-degrading bacteria, *Flavobacterium* (O’Reilly and Crawford, 1989), *Novosphingobium* (Tiirola et al., 2002), *Sphingopyxis* (Xu et al., 2015), their relative abundances significantly increased in FLB community, while *Labrys* (Carvalho et al., 2005) was more abundant in PAB community. Many strains from *Sphingopyxis* have microcystin-degrading ability (Shimizu et al., 2012; Xu et al., 2015). As a water-soluble compound, microcystin released by *Microcystis* can easily diffuse into surrounding water and could be utilized by microcystin-degraders, e.g., *Sphingopyxis*. In PAB community, the relative abundance of some potentially methanol-utilizing [i.e., *Phreatobacter* (Tóth et al., 2014)] and ferrous iron-oxidizing genera [i.e., *Ferroviario* (Dubinina and Sorokina, 2014)] also significantly increased. In FLB community, the relative abundance of genera known for algal-derived DOC-utilizing (e.g., *Limnohabitans* and *Limnobacter*), eutrophic water-affiliating [e.g., *CL500-29 marine group* and *hgcI clade* (Lindh et al., 2015)] and pathogenic microorganism [e.g., *Flavobacterium* (Nicolas et al., 2008), *Legionella* (Hägele et al., 2000), *Mycobacterium* (Cosma et al., 2003)] significantly increased. The physiology of many other genera remains unclear (e.g., *Hirschia, Candidatus Trichorickettsia* and so on).

Ecosystems are very complex, with complicated networks that form between considerably interacting organisms (Montoya et al., 2006). In this study, a network-based approach using RMT was used to show the co-occurrence pattern and delineated interactions of bacterial communities during cyanobacterial blooms. The specific microbial interaction networks of PAB and FLB were established, and the role of cyanobacteria (especially for *M. aeruginosa*) in the network was proposed.

The stability of an ecosystem function lies in species interactions (Zhou et al., 2010). Lower connectivity in a community leads to higher functional stability of the system (e.g., scale-free network) because the whole network module will be less affected by the loss of nodes. (Zhou et al., 2010). For PAB, the higher connectively (avgK), highly concentrated modularity and fewer nodes could result in greater vulnerability than that of FLB. For FLB, the lower connectivity (avgK), higher network size and higher bacterial diversity could result in greater resilience to disturbances than that of PAB. The differences in topological features (e.g., connectivity and geodesic distance) of PAB and FLB networks could be associated with the spatial distances among bacteria, i.e., bacteria on particles are overall closer to each other than FLB. Accordingly, direct interactions, such as mutualism, competition or predation are more likely to occur in the PAB community. Taxa tended to covary in both PAB and FLB networks with predominantly positive correlations (>88%), indicting similarly preferred conditions or cooperative behaviors (e.g., cross feeding, syntrophic interactions and mutualistic interactions) of most bacteria during *M. aeruginosa* bloom (Raes and Bork, 2008).

In an ecological network, module hubs and connectors play crucial roles in the maintenance of community stability and are considered keystone species in the system (Zhou et al., 2010; Shi et al., 2016). The putative keystone nodes in PAB and FLB networks were lowly abundant, which indicates that low abundance taxa may play important roles in maintaining network structures in freshwater microbial communities. This finding is consistent with studies of soil microbial communities (Lupatini et al., 2014; Shi et al., 2016). Alphaproteobacteria was the most important group in the PAB network and occupied the three module centers: *Meganema, Pleomorphomonas* and *Brevundimonas* sp.. For the *Meganema* genus, *M. perideroedes* is the sole described species, which was found to be a versatile facultative anaerobe in activated sludge with ability to utilize various organic substrates in aerobic conditions and use nitrate and nitrite as electron acceptors (Kragelund et al., 2005), and McIlroy et al. (2015) thought it is a potentially facultative methylotrophy based on the genome of stain Gr1; for *Pleomorphomonas*, almost all the species of this genus are N-fixing bacteria, i.e., *P. diazotrophica, P. diazotrophica*, and *P. oryzae* (Xie and Yokota, 2005; Im et al., 2006; Madhaiyan et al., 2013), and *Pleomorphomonas* sp. were found can methylate Hg (II) (Achá et al., 2012); *Brevundimonas* sp. have been found the abilities of algicidal effect on *M. aeruginosa*, growth promotion on *Chlorella ellipsoide*, cadmium biosorption, lactofen-hydrolyzing, quinoline-degrading and antimicrobial silver nanoparticles producing (Park et al., 2008; Masoudzadeh et al., 2011; Rajamanickam et al., 2013; Wang C. et al., 2015).

For the five identified genera of module hubs and connectors in FLB network, *Nitrospira* are known as the most diverse and widespread nitrifiers in various ecosystems, e.g., natural environments and biological wastewater treatment plants (Lücker et al., 2010; Feng et al., 2016; Pinto et al., 2016). Ecological function or relationships with algal blooms of the remaining four genera are unclear except for the following information: for *Fluviicola*, only novel species descriptions have been reported and they were mainly isolated from fresh water and chemical industrial wastewater (O’Sullivan et al., 2005; Yang et al., 2014); *CL500-29 marine group* is widespread in waters, particularly in copiotrophic environments (Lindh et al., 2015); *Legionella* are wide-spread pathogens of human, animals, protozoa and aquatic eukaryotes (Golovlev, 2000; Hägele et al., 2000), and most species are aquatic origin; *C. catalasitica* is the sole species of *Crocinitomix*, which was isolated from algal-rich littoral zone of Auke Bay, AK, United States (Bowman et al., 2003). In general, module hubs of both networks were potentially involved in biogeochemical cycling of nitrogen taxa. This finding indicates that bacteria with ecological physiological functions of N metabolism might be quite important in the two communities. Moreover, module I of PAB network was centered with a potentially algicidal bacteria of *M. aeruginosa* (OTU1156, *Brevundimonas*), which indicates that algicidal bacteria might have important roles in PAB communities.

As the dominant phytoplankton phylum in Dianchi Lake, cyanobacteria (green nodes in **Figure 5**) were mostly connected with negative links (especially for the node OTU359), representing antagonistic relationships such as predator-prey, competition or variable fitness (Chow et al., 2014). As the photosynthetic organism, phytoplankton is often the most important biotic importer of organic substrates (e.g., DOM) in water systems. Cyanobacteria could act as a nutrient contributor by supplying heterotrophic bacteria with organic substrates from photosynthesis or by acting as prey (Paver et al., 2013).

The cyanobacteria node OTU359 represents the bloom-causing species *M. aeruginosa*, which is the most common colonial cyanobacteria in fresh water. As an oxygenic and photoautotrophic species, *M. aeruginosa* often form blooms in eutrophic waters and produce toxic microcystins. OTU359 was the only node with purely negative connections in module I, indicating its uniquely antagonistic relationships with neighboring nodes. It had 18 neighboring nodes in the PAB network, including the potentially algicial bacteria OTU1156 (*Brevundimonas*), which was the hub of module I. Among other neighbors of OTU359, OTU423-*CL500*-*29 marine group* are widespreadly aquatic bacteria; OTU1032-*Gemmatimonas* strains have photosynthetic pigments [i.e., bacteriochlorophyll a (Zeng et al., 2015) and carotenoids (Takaichi et al., 2009)] and polyphosphate-accumulating functions (Zhang et al., 2003). It is difficult to understand the connection of other neighbors with OTU359 because their ecophysiology is unclear (e.g., *SM1A02, I-10*) or appears irrelevant to cyanobacteria [e.g., *Phenylobacterium*; the physiology of reported strains are mainly involved in organic contaminant biodegradation (Tiago et al., 2005; Eberspächer and Lingens, 2006)]. The relationships between OTU359 and the related two neighbors (OTU74 and OTU 190) in the FLB network were also unclear.

The bloom-causing species *M. aeruginosa* (OTU359) was assumed to play different roles in PAB and FLB communities according to its network topological characteristics. Although node OTU 359 has few connections (two links) within the major module of the FLB network, *M. aeruginosa* seems more important because it connected to the hub of the largest module (module I) and occupied a higher hierarchical position with the 18 links in the PAB network. Therefore, *M. aeruginosa* possibly played a more important role in PAB communities than that of FLB communities. Additionally, the tight connections between nodes in the PAB network could indicate quick and effective metabolic interactions (Zhou et al., 2010) among species by transferring considerable organic matter from algae quickly in this phycosphere, thus indicating their potentially important roles in material and energy flows in this aquatic ecosystem.

In general, the communities of PAB and FLB during a *M. aeruginosa* bloom were significantly different in their structure, diversity, microbial interaction network and the topological role of *M. aeruginosa*. For PAB network, bacterial connections were tighter, potentially indicating more efficient metabolism interactions than that of FLB network; the higher importance of *M. aeruginosa* (OTU359) was indicated by high connectivity in the core region of the PAB network; Alphaproteobacteria were predominant, among which potentially nitrogen-fixing and algicidal bacteria occupied the module centers. FLB communities had significantly higher diversity and were sensitive to the detected environmental factors; genera related to methane-utilizing and pathogenic bacteria had significantly higher relative abundance; *M. aeruginosa* was marginal in the network, with less connectivity and more modules and nodes, possibly leading to the higher resilience of the ecological function than that of PAB. In both PAB and FLB interaction networks, a co-occurrence pattern was predominant, with positive connections. Bacteria affiliated with N metabolism were important by occupying the module hubs. Here, we have made an important attempt to explore the differential response of PAB and FLB communities to a cyanobacterial bloom by applying network-based analyses. Our findings provide ecological insights into the different interactions among PAB or FLB communities during algal blooms.

## Author Contributions

YI and FL designed the research and revised the manuscript. CY, QW, PS, JL, and JK analyzed the data and drafted the manuscript. CY, QW, PS, JL, LL, XD, XZ, and XP contributed to the sampling and experiments. LL, XD, XZ, and XP revised the manuscript. All authors approved the final manuscript and agreed to be responsible for all aspects of the work.

## Conflict of Interest Statement

The authors declare that the research was conducted in the absence of any commercial or financial relationships that could be construed as a potential conflict of interest.

## Footnotes

1 http://drive5.com/uparse/

2 http://rdp.cme.msu.edu/

3 http://ieg2.ou.edu/MENA

## Supplementary Material

FIGURE S1 The number of identified taxa at different levels based on total sequences **(a)**, the Venn diagrams **(b)** for taxa number of PAB (gray-tinted cycles with dashed) and FLB (white cycles with solid line).

FIGURE S2 Distribution of bacterial taxa at phylum level based on sequence data of Resample 1. Sample names with a and b represent the PAB and FLB, respectively.
