# Supplementary figures and images for: Distinct Network Interactions in Particle-Associated and Free-Living Bacterial Communities during a Microcystis aeruginosa Bloom in a Plateau Lake
Source: Front Microbiol. 2017 Jun 30;8:1202. doi: 10.3389/fmicb.2017.01202 (PMC5492469; doi:10.3389/fmicb.2017.01202)

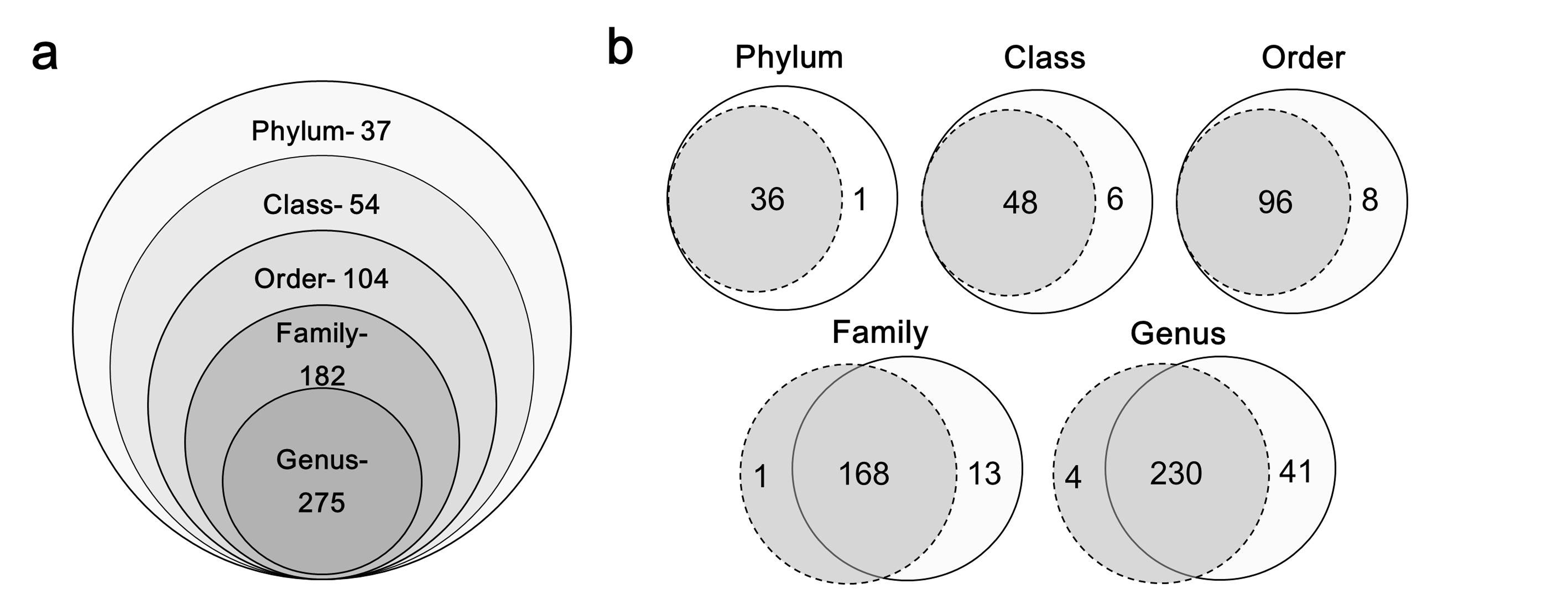

Supplement: FIGURE S1 — The number of identified taxa at different levels based on total sequences (a), the Venn diagrams (b) for taxa number of PAB (gray-tinted cycles with dashed) and FLB (white cycles with solid line). [file Image_1.TIF]

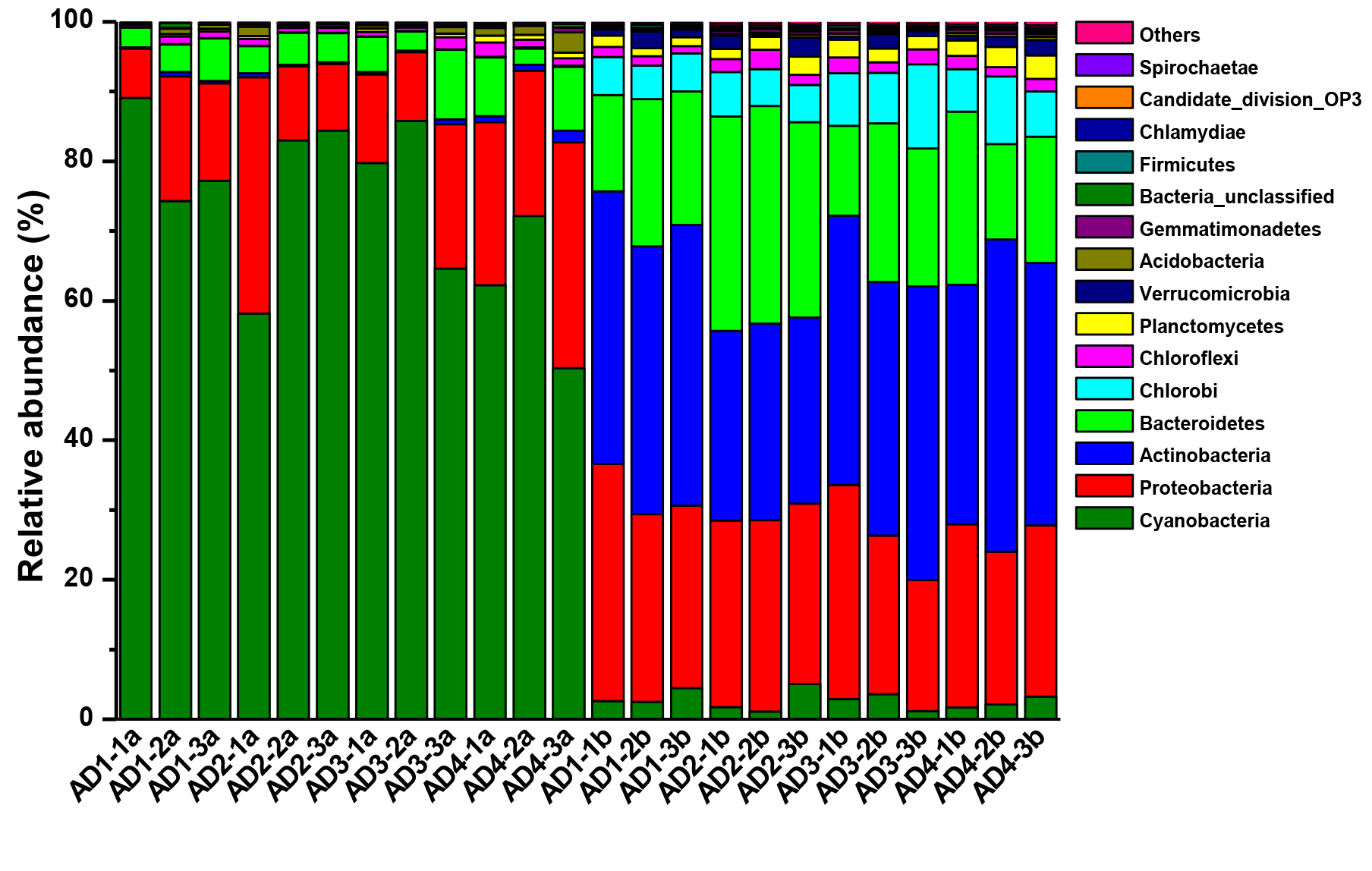

Supplement: FIGURE S2 — Distribution of bacterial taxa at phylum level based on sequence data of Resample 1. Sample names with a and b represent the PAB and FLB, respectively. [file Image_2.TIF]
