# Supplementary material for: Distinct Network Interactions in Particle-Associated and Free-Living Bacterial Communities during a Microcystis aeruginosa Bloom in a Plateau Lake
Source: Front Microbiol. 2017 Jun 30;8:1202. doi: 10.3389/fmicb.2017.01202 (PMC5492469; doi:10.3389/fmicb.2017.01202)
Supplement: Supplementary file 3 [file Data_Sheet_1.docx]

Table S1 Algal distribution at phylum and genus levels based on microscopic examination

| **Sample** |  | **Cyanobacteria** |  | **Bacillariophyta** | | |  | **Pyrropphyta** | **Unidentified microalgae** |
| --- | --- | --- | --- | --- | --- | --- | --- | --- | --- |
|  |  | ***Microcystis*** |  | ***Cyclotella*** | ***Synedra*** | ***Coscinodiscus*** |  | ***Ceratium*** |  |
| AD1-1 |  | 99.86 |  | 0 | 0 | 0 |  | 0.07 | 0.07 |
| AD1-2 |  | 100.00 |  | 0 | 0 | 0 |  | 0 | 0 |
| AD1-3 |  | 99.72 |  | 0.09 | 0 | 0 |  | 0 | 0.19 |
| AD2-1 |  | 98.52 |  | 0 | 0 | 0 |  | 0 | 1.48 |
| AD2-2 |  | 98.38 |  | 0 | 0 | 0 |  | 0 | 1.62 |
| AD2-3 |  | 99.08 |  | 0 | 0.12 | 0.46 |  | 0 | 0.35 |
| AD3-1 |  | 98.30 |  | 0 | 0 | 0.57 |  | 0 | 1.14 |
| AD3-2 |  | 98.50 |  | 0 | 0 | 0 |  | 0 | 1.50 |
| AD3-3 |  | 99.75 |  | 0.05 | 0 | 0 |  | 0 | 0.20 |
| AD4-1 |  | 98.27 |  | 0 | 0 | 0 |  | 0 | 1.73 |
| AD4-2 |  | 99.71 |  | 0.14 | 0 | 0 |  | 0 | 0.14 |
| AD4-3 |  | 98.63 |  | 0 | 0 | 0.68 |  | 0 | 0.68 |

Table S2 Summary of sequencing data (V3–V4 region of microbial 16S rRNA gene) for all samples.

| **Sample** |  | **Original** | | | | | |  |  | **Resampling 1** | | |  | **Resampling 2** | | |
| --- | --- | --- | --- | --- | --- | --- | --- | --- | --- | --- | --- | --- | --- | --- | --- | --- |
|  |  | **Read No.** | **OTU No.** | **Chao value** | **Coverage (%)** | **Shannon**  **diversity** |  |  | **Read No.** | | **OTU No.** | **Shannon**  **diversity** |  | **Read No.** | **OTU No.** | **Shannon**  **diversity** |
| **AD1-1a** |  | 99733 | 502 | 734 | 99.8 | 1 |  |  | 31736 | | 317 | 0.97 |  | 4512 | 311 | 4.13 |
| **AD1-2a** |  | 70579 | 601 | 835 | 99.7 | 1.91 |  |  |  |  | 434 | 1.88 |  |  | 330 | 4.28 |
| **AD1-3a** |  | 77224 | 585 | 766 | 99.8 | 1.79 |  |  |  |  | 417 | 1.78 |  |  | 318 | 4.23 |
| **AD2-1a** |  | 45514 | 490 | 731 | 99.6 | 2.36 |  |  |  |  | 401 | 2.34 |  |  | 268 | 3.88 |
| **AD2-2a** |  | 67549 | 506 | 658 | 99.8 | 1.39 |  |  |  |  | 370 | 1.34 |  |  | 328 | 4.26 |
| **AD2-3a** |  | 73976 | 500 | 752 | 99.8 | 1.31 |  |  |  |  | 355 | 1.28 |  |  | 308 | 4.18 |
| **AD3-1a** |  | 72885 | 619 | 878 | 99.7 | 1.63 |  |  |  |  | 462 | 1.6 |  |  | 352 | 4.35 |
| **AD3-2a** |  | 31736 | 382 | 543 | 99.5 | 1.17 |  |  |  |  | 368 | 1.17 |  |  | 338 | 4.3 |
| **AD3-3a** |  | 75548 | 706 | 903 | 99.7 | 2.46 |  |  |  |  | 520 | 2.43 |  |  | 345 | 4.34 |
| **AD4-1a** |  | 78583 | 784 | 932 | 99.8 | 2.63 |  |  |  |  | 564 | 2.6 |  |  | 391 | 4.55 |
| **AD4-2a** |  | 71734 | 605 | 814 | 99.7 | 1.98 |  |  |  |  | 445 | 1.94 |  |  | 324 | 4.24 |
| **AD4-3a** |  | 98344 | 802 | 980 | 99.8 | 2.94 |  |  |  |  | 537 | 2.91 |  |  | 333 | 4.23 |
| **AD1-1b** |  | 78634 | 1067 | 1262 | 99.7 | 4.67 |  |  |  |  | 785 | 4.63 |  |  | 414 | 4.5 |
| **AD1-2b** |  | 70079 | 1062 | 1255 | 99.7 | 4.9 |  |  |  |  | 797 | 4.85 |  |  | 443 | 4.7 |
| **AD1-3b** |  | 67001 | 1047 | 1284 | 99.6 | 4.75 |  |  |  |  | 788 | 4.7 |  |  | 421 | 4.51 |
| **AD2-1b** |  | 54669 | 1038 | 1252 | 99.6 | 5.22 |  |  |  |  | 850 | 5.19 |  |  | 475 | 5.04 |
| **AD2-2b** |  | 57683 | 1023 | 1304 | 99.6 | 5.15 |  |  |  |  | 822 | 5.12 |  |  | 436 | 4.96 |
| **AD2-3b** |  | 65744 | 1049 | 1218 | 99.7 | 5.2 |  |  |  |  | 822 | 5.17 |  |  | 471 | 5 |
| **AD3-1b** |  | 56126 | 982 | 1180 | 99.6 | 4.79 |  |  |  |  | 788 | 4.78 |  |  | 424 | 4.64 |
| **AD3-2b** |  | 61747 | 1088 | 1257 | 99.6 | 4.97 |  |  |  |  | 865 | 4.94 |  |  | 473 | 4.82 |
| **AD3-3b** |  | 59909 | 991 | 1162 | 99.7 | 4.48 |  |  |  |  | 816 | 4.49 |  |  | 436 | 4.36 |
| **AD4-1b** |  | 69143 | 1110 | 1360 | 99.7 | 5.09 |  |  |  |  | 864 | 5.06 |  |  | 502 | 4.91 |
| **AD4-2b** |  | 66033 | 1064 | 1236 | 99.7 | 4.56 |  |  |  |  | 827 | 4.56 |  |  | 446 | 4.44 |
| **AD4-3b** |  | 68554 | 1119 | 1262 | 99.7 | 4.99 |  |  |  |  | 871 | 4.95 |  |  | 463 | 4.79 |

Sample names with –a and -b represent the PAB and FLB, respectively. Resample 1/2 were based on the total data set/ the data set after removing cyanobacterial sequences, respectively.

Table S3 The relative abundance of dominant bacterial phyla, genera and species of all samples based on Resample 1.

| **Sample** | **Phylum (%)** | | | |  | **Genus (%)** | | |  | **Species (%)** | | |
| --- | --- | --- | --- | --- | --- | --- | --- | --- | --- | --- | --- | --- |
|  | **Cya.** | **Pro.** | **Act.** | **Bac.** |  | ***Mic.*** | ***H.C.*** | ***C.M.G.*** |  | ***M. a.*** (OTU359) | ***Act.Un1*** (OTU674) | ***Act.Un2*** (OTU512) |
| **AD1-1a** | 89.11 | 7.04 | 0.20 | 2.85 |  | 86.14 | 0.01 | 0.07 |  | 86.14 | < 0.01 | 0.02 |
| **AD1-2a** | 74.33 | 17.86 | 0.60 | 3.97 |  | 70.65 | <0.01 | 0.33 |  | 70.65 | 0 | 0.19 |
| **AD1-3a** | 77.23 | 13.98 | 0.34 | 6.09 |  | 71.80 | < 0.01 | 0.14 |  | 71.80 | 0 | 0.03 |
| **AD2-1a** | 58.16 | 33.87 | 0.61 | 3.89 |  | 57.61 | 0.01 | 0.20 |  | 57.61 | < 0.01 | 0.06 |
| **AD2-2a** | 83.00 | 10.60 | 0.27 | 4.57 |  | 79.77 | 0.01 | 0.06 |  | 79.77 | < 0.01 | 0.02 |
| **AD2-3a** | 84.39 | 9.59 | 0.20 | 4.23 |  | 80.85 | < 0.01 | 0.08 |  | 80.85 | < 0.01 | 0.02 |
| **AD3-1a** | 79.74 | 12.71 | 0.37 | 5.05 |  | 76.29 | 0.01 | 0.14 |  | 76.29 | < 0.01 | 0.04 |
| **AD3-2a** | 85.77 | 9.88 | 0.22 | 2.76 |  | 83.31 | 0.01 | 0.09 |  | 83.31 | < 0.01 | 0.02 |
| **AD3-3a** | 64.63 | 20.68 | 0.70 | 9.99 |  | 59.66 | 0.01 | 0.26 |  | 59.66 | < 0.01 | 0.05 |
| **AD4-1a** | 62.21 | 23.42 | 0.84 | 8.43 |  | 58.71 | 0.00 | 0.35 |  | 58.71 | < 0.01 | 0.10 |
| **AD4-2a** | 72.18 | 20.79 | 0.87 | 2.37 |  | 69.39 | 0.01 | 0.33 |  | 69.39 | < 0.01 | 0.17 |
| **AD4-3a** | 50.37 | 32.34 | 1.67 | 9.20 |  | 48.89 | 0.01 | 0.58 |  | 48.89 | 0 | 0.14 |
| **AD1-1b** | 2.60 | 34.00 | 39.08 | 13.82 |  | 0.22 | 22.19 | 8.40 |  | 0.22 | 7.23 | 4.58 |
| **AD1-2b** | 2.48 | 26.95 | 38.40 | 21.11 |  | 0.09 | 22.25 | 8.04 |  | 0.09 | 6.48 | 4.41 |
| **AD1-3b** | 4.46 | 26.16 | 40.25 | 19.14 |  | 0.12 | 26.09 | 6.98 |  | 0.12 | 9.62 | 3.68 |
| **AD2-1b** | 1.73 | 26.75 | 27.21 | 30.73 |  | 0.36 | 14.90 | 6.87 |  | 0.36 | 4.35 | 4.44 |
| **AD2-2b** | 1.12 | 27.41 | 28.23 | 31.18 |  | 0.21 | 10.11 | 10.37 |  | 0.21 | 3.02 | 7.62 |
| **AD2-3b** | 5.06 | 25.86 | 26.65 | 28.06 |  | 0.67 | 13.51 | 7.48 |  | 0.67 | 4.20 | 5.00 |
| **AD3-1b** | 2.90 | 30.71 | 38.62 | 12.88 |  | 0.42 | 17.77 | 12.84 |  | 0.42 | 5.31 | 8.70 |
| **AD3-2b** | 3.61 | 22.72 | 36.37 | 22.76 |  | 0.37 | 21.08 | 9.74 |  | 0.37 | 7.40 | 6.72 |
| **AD3-3b** | 1.14 | 18.82 | 42.08 | 19.77 |  | 0.21 | 31.43 | 6.36 |  | 0.21 | 11.27 | 4.49 |
| **AD4-1b** | 1.70 | 26.29 | 34.30 | 24.85 |  | 0.08 | 16.28 | 12.03 |  | 0.08 | 7.17 | 9.44 |
| **AD4-2b** | 2.16 | 21.84 | 44.77 | 13.71 |  | 0.11 | 27.24 | 12.06 |  | 0.11 | 10.72 | 8.84 |
| **AD4-3b** | 3.24 | 24.53 | 37.65 | 18.08 |  | 0.18 | 16.03 | 15.30 |  | 0.18 | 5.22 | 10.43 |

Cya.: Cyanobacteria, Pro.: Proteobacteria, Act.: Actinobacteria, Bac.: Bacteroidetes, Mic.: *Microcystis* H.C.: hgcI clade of Actinobacteria, C.M.G.: CL500-29 marine group of Actinobacteria , M. a.: *Microcystis* *aeruginosa*, Act.Un1: uncultured species of hgcI clade, Act.Un2: unclassified species of CL500-29 marine group. OTU359, OTU674 and OTU512 are the OTUs of the three dominant species, respectively. The table was based on resampled sequences (31735 reads), sample names with -a and -b represent the PAB and FLB, respectively.

| **Phylum** | **Relative abundance (%)** | | ***P* value (T-test)** |
| --- | --- | --- | --- |
|  | **PAB** | **FLB** |  |
| Proteobacteria | 65.88 ± 6.34 | 26.77 ± 4.27 | < 0.01 |
| Chloroflexi | 3.83 ± 1.06 | 1.72 ± 0.45 | < 0.01 |
| Acidobacteria | 2.96 ± 1.15 | 0.33 ± 0.16 | < 0.01 |
| Actinobacteria | 1.96 ± 0.70 | 37.43 ± 5.72 | < 0.01 |
| Unclassified bacteria | 0.87 ± 0.68 | 0.20 ± 0.10 | < 0.01 |
| Gemmatimonadetes | 0.69 ± 0.27 | 0.40 ± 0.13 | < 0.01 |
| Chlorobi | 0.22 ± 0.11 | 6.87 ± 2.19 | < 0.01 |
| SHA-109* | 0.13 ± 0.07 | 0.03 ± 0.03 | < 0.01 |
| Verrucomicrobia | 0.09 ± 0.08 | 1.40 ± 0.94 | < 0.01 |
| Chlamydiae | 0.02 ± 0.02 | 0.24 ± 0.14 | < 0.01 |
| OP3* | 0.01 ± 0.02 | 0.11 ± 0.10 | < 0.01 |
| Saccharibacteria | 0 | 0.03 ± 0.03 | 0.03 |
| TM6* | 0 | 0.08 ± 0.07 | < 0.01 |
| Deinococcus-Thermus | 0 | 0.04 ± 0.03 | < 0.01 |

Table S4 Relative abundance of significantly changed phyla in PAB and FLB communities.

Table S5 The genera with significantly higher (A) and lower (B) relative abundance in PAB when compared to FLB.

A B

| **Genus** | **Relative abundance** | | ***P*** |  | **Genus** | **Relative abundance** | | ***P*** |
| --- | --- | --- | --- | --- | --- | --- | --- | --- |
|  | **PAB** | **FLB** |  |  |  | **PAB** | **FLB** |  |
| *Ferrovibrio* | 0.05 | 0 | < 0.01 |  | *Acidibacter* | 0.84 | 1.352 | < 0.01 |
| *Nannocystaceae uncultured* | 0.007 | 0 | 0.04 |  | *Methylocaldum* | 0.018 | 0.041 | 0.04 |
| *OM27 clade* | 4.778 | 0.046 | < 0.01 |  | *Haliscomenobacter* | 0.063 | 0.144 | < 0.01 |
| *Reyranella* | 4.536 | 0.048 | < 0.01 |  | *Sporichthya* | 0.018 | 0.044 | 0.02 |
| *Bryobacter* | 2.475 | 0.066 | < 0.01 |  | *Romboutsia* | 0.022 | 0.057 | < 0.01 |
| *Phreatobacter* | 0.039 | 0.002 | < 0.01 |  | *Hirschia* | 0.07 | 0.187 | < 0.01 |
| *Bradyrhizobium* | 0.22 | 0.013 | < 0.01 |  | *Caulobacter* | 0.072 | 0.194 | < 0.01 |
| *Aquimonas* | 0.177 | 0.011 | < 0.01 |  | *Porphyrobacter* | 0.068 | 0.188 | < 0.01 |
| *Labrys* | 0.175 | 0.013 | < 0.01 |  | *Hyphomonas* | 0.126 | 0.362 | < 0.01 |
| *Phaselicystis* | 0.395 | 0.033 | < 0.01 |  | *CL500-3* | 0.404 | 1.524 | < 0.01 |
| *Crocinitomix* | 0.109 | 0.009 | < 0.01 |  | *Flavobacterium* | 0.427 | 1.803 | < 0.01 |
| *Silanimonas* | 2.017 | 0.192 | < 0.01 |  | *Candidatus Microthrix* | 0.02 | 0.111 | < 0.01 |
| *Candidatus Competibacter* | 0.018 | 0.002 | 0.02 |  | *Sandaracinobacter* | 0.004 | 0.022 | 0.02 |
| *Pleomorphomonas* | 0.017 | 0.002 | 0.04 |  | *BD1-7 clade* | 0.042 | 0.26 | < 0.01 |
| *Meganema* | 0.05 | 0.006 | < 0.01 |  | *Candidatus Captivus* | 0.02 | 0.126 | < 0.01 |
| *Inhella* | 0.558 | 0.066 | < 0.01 |  | *Malikia* | 0.009 | 0.057 | < 0.01 |
| *Candidatus Odyssella* | 0.046 | 0.006 | < 0.01 |  | *Candidatus Aquirestis* | 0.083 | 0.519 | < 0.01 |
| *Tepidicella* | 0.388 | 0.057 | < 0.01 |  | *Legionella* | 0.018 | 0.129 | < 0.01 |
| *Sandaracinus* | 0.037 | 0.006 | < 0.01 |  | *Parvibaculum* | 0.002 | 0.013 | 0.03 |
| *Crenothrix* | 0.024 | 0.004 | 0.02 |  | *Polynucleobacter* | 0.059 | 0.469 | < 0.01 |
| *Bdellovibrio* | 0.874 | 0.144 | < 0.01 |  | *Mycobacterium* | 0.124 | 1.127 | < 0.01 |
| *Candidatus Trichorickettsia* | 0.011 | 0.002 | 0.03 |  | *Arenimonas* | 0.026 | 0.246 | < 0.01 |
| *Pedomicrobium* | 0.183 | 0.031 | < 0.01 |  | *Methylomonas* | 0.002 | 0.02 | 0.02 |
| *Turneriella* | 0.042 | 0.007 | < 0.01 |  | *Alpinimonas* | 0.002 | 0.02 | 0.02 |
| *Roseomonas* | 2.527 | 0.558 | < 0.01 |  | *CL500-29 marine group* | 0.774 | 9.925 | < 0.01 |
| *Rheinheimera* | 0.153 | 0.05 | < 0.01 |  | *Novosphingobium* | 0.004 | 0.057 | 0.04 |
| *Gemmatimonas* | 0.604 | 0.251 | < 0.01 |  | *Lacibacter* | 0.022 | 0.366 | < 0.01 |
| *Lautropia* | 0.129 | 0.055 | < 0.01 |  | *Ferruginibacter* | 0.006 | 0.1 | < 0.01 |
| *Woodsholea* | 0.179 | 0.096 | < 0.01 |  | *Wandonia* | 0.009 | 0.264 | < 0.01 |
| *SM1A02* | 0.53 | 0.375 | 0.02 |  | *Filimonas* | 0.004 | 0.137 | < 0.01 |
|  |  |  |  |  | *Chitinophaga* | 0.011 | 0.464 | < 0.01 |
|  |  |  |  |  | *Limnohabitans* | 0.063 | 3.097 | 0.02 |
|  |  |  |  |  | *Pseudospirillum* | 0.002 | 0.1 | < 0.01 |
|  |  |  |  |  | *Pedobacter* | 0.002 | 0.102 | < 0.01 |
|  |  |  |  |  | *Paenibacillus* | 0.002 | 0.111 | < 0.01 |
|  |  |  |  |  | *Fluviicola* | 0.02 | 1.234 | < 0.01 |
|  |  |  |  |  | *Paucimonas* | 0.011 | 0.796 | < 0.01 |
|  |  |  |  |  | *MWH-UniP1 aquatic group* | 0.018 | 1.49 | < 0.01 |
|  |  |  |  |  | *Limnobacter* | 0.002 | 0.157 | < 0.01 |
|  |  |  |  |  | *Sphaerotilus* | 0.004 | 0.462 | < 0.01 |
|  |  |  |  |  | *Roseiflexus* | 0.009 | 1.204 | < 0.01 |
|  |  |  |  |  | *LD28 freshwater group* | 0.009 | 1.213 | < 0.01 |
|  |  |  |  |  | *Ramlibacter* | 0.002 | 0.29 | < 0.01 |
|  |  |  |  |  | *Sphingopyxis* | 0.002 | 0.382 | < 0.01 |
|  |  |  |  |  | *Candidatus Aquiluna* | 0.002 | 1.044 | < 0.01 |
|  |  |  |  |  | *hgcI clade* | 0.022 | 20.754 | < 0.01 |
|  |  |  |  |  | *Candidatus Planktophila* | 0 | 0.083 | < 0.01 |
|  |  |  |  |  | *Candidatus Methylacidiphilum* | 0 | 0.054 | < 0.01 |
|  |  |  |  |  | *Zymomonas* | 0 | 0.042 | < 0.01 |
|  |  |  |  |  | *12up* | 0 | 0.035 | 0.03 |
|  |  |  |  |  | *PRD01a011B* | 0 | 0.035 | 0.02 |
|  |  |  |  |  | *Meiothermus* | 0 | 0.031 | < 0.01 |
|  |  |  |  |  | *Owenweeksia* | 0 | 0.031 | < 0.01 |
|  |  |  |  |  | *Aeromonas* | 0 | 0.02 | < 0.01 |
|  |  |  |  |  | *Emticicia* | 0 | 0.017 | 0.01 |
|  |  |  |  |  | *Cloacibacterium* | 0 | 0.013 | 0.01 |
